# Soil bacterial community composition in rice-turtle coculture systems with different planting years

**DOI:** 10.1038/s41598-023-49701-1

**Published:** 2023-12-19

**Authors:** Ren Wang, Weiwei Ma, Dan Wu, Yin Zhang, Xuehu Ma, Guangdong Lv, Jiaolong Ding, Zhiqiang Fu, Can Chen, Huang Huang

**Affiliations:** 1 College of Agronomy, Hunan Agricultural University, Changsha, 410128 China; 2 Hunan Engineering Research Center of Rice Field Ecological Planting and Breeding, Changsha, 410128 China; 3 Yueyang Agricultural and Rural Affairs Center, Yueyang, 414004 China; 4 Hengyang Academy of Agricultural Sciences, Hengyang, 421100 China

**Keywords:** Ecology, Biodiversity

## Abstract

The rice-turtle coculture system is the most special rice-fish integrated farming system. In this study, we selected four paddy fields, including a rice monoculture paddy and three rice-turtle paddies with different planting years, to investigate the soil bacterial community composition with Illumina MiSeq sequencing technology. The results indicated that the contents of soil available nitrogen (AN), soil available phosphorus (AP) and soil organic matter (OM) in 9th year of rice-turtle paddy (RT9) were increased by 5.40%, 51.11% and 23.33% compared with rice monoculture paddy (CK), respectively. Significant differences of *Acidobacteria, Desulfobacteria, Crenarchaeota* were observed among the different rice farming systems. The relative abundance of *Methylomonadaceae*, *Methylococcaceae* and *Methylophilaceae* in RT9 was significantly higher than that in other treatments. RT9 had significantly lower relative abundance of *Acidobacteria*, but significantly higher relative abundance of *Proteobacteria* than other treatments. Redundancy analysis showed that soil AN and AP contents were the major factors influencing the abundance of the dominant microbes, wherein *Methylomonadaceae, Methylococcaceae* and *Methylophilaceae* were positively correlated with OM. The findings revealed the rice-turtle coculture system in the 9th year had higher soil nutrients and soil bacterial diversity, but there was also a risk of increasing methane emissions.

## Introduction

The rice-fish coculture systems originated in China, with the earliest record dated back to 1700 years ago^1^. In 2021, the area of rice–fish systems in China is 2.64 × 10^6^ ha, which accounts for approximately 8.58% of the total rice planting area in China^2,3^. Rice-turtle coculture system is the most unique pattern in rice-fish integrated farming system. The soft-shelled turtle is an amphibious temperature-changing animal, which has wide feeding habits, strong stress resistance and self-reproduction ability, and can live in paddy fields all year round. The rice-turtle co-culture system has long symbiotic period, strong anti-risk ability, high quality products, high economic and ecological benefits^4^.

Microorganism is an important part of soil system that plays crucial roles in the circulation of soil nutrients and biochemical reactions^5,6^. The quantity and community composition of the soil bacteria are mainly affected by environmental factors such as soil nutrients, soil aeration, pH and so on^7^. Thus, the soil microbial community can be used as an indicator to track changes in various land management methods, such as tracking changes in restoration outcomes^8^. Many studies have demonstrated that the rice-fish integrated farming patterns could increase the number and community diversity of soil microorganisms^9–11^, especially bacteria that play a crucial role in soil carbon and nitrogen mineralization^12^. *Proteobacteria, Bacteroidetes, Acidobacteria,* and *Chloroflexi* were generally dominant phyla in the paddy soil. However, more studies payed attention to the environmental factors in different rice-fish coculture systems, such as soil properites, that affect the changes in soil microbial community. Will soil nutrients and soil bacterial diversity increasing in response to longer planting years in the rice-turtle coculture systems?

In this study, the evolutionary characteristics of the soil bacterial communities in the rice-turtle coculture systems with different planting years were investigated using Illumina MiSeq sequencing. Our hypothesis is that with soil nutrients increasing in response to longer planting years in the rice-turtle coculture systems, and the soil bacterial community structure changed significantly. These differentiations of soil bacterial community may relate to some soil factors or crucial taxa. To test this hypothesis, a rice monoculture paddy and 3 rice turtle paddies with different planting years (3 years, 6 years and 9 years) on Kongpuzhong Family Farm were chosen to investigate the soil bacterial community composition.

## Results

### Soil properties

The soil properties of the four treatments were shown in Table 1. The contents of AN and AP were significantly different among treatments. The highest soil AN and AP content was observed in RT9, which was significantly higher than that in CK, with the increase of 25.40% and 51.11% respectively. There was no significant difference in AK content among treatments. The soil organic matter content of RT9 treatments was significantly higher than that of CK treatment, with the increase of 23.33%. The soil pH of the rice-turtle coculture systems was higher than that of CK, and there were significantly different among CK, RT3 and RT6. The results showed that the rice-turtle coculture could effectively reduce soil acidity, and significantly increase soil available nitrogen, available phosphorus and organic matter content when the planting years reached 9 years.

**Table 1:** Soil properties in different rice system.

Treatment	AN (mg/kg)	AP (mg/kg)	AK (mg/kg)	OM (g/kg)	pH
CK	115.73 ± 0.81c	34.36 ± 1.32c	70.00 ± 2.65a	21.60 ± 0.86b	5.29 ± 0.20c
RT3	135.33 ± 7.18b	25.05 ± 0.52d	70.00 ± 5.29a	25.50 ± 0.29a	5.46 ± 0.02b
RT6	107.33 ± 1.62d	43.77 ± 1.34b	66.67 ± 5.77a	21.78 ± 0.69b	5.63 ± 0.03a
RT9	145.13 ± 4.90a	51.92 ± 4.11a	63.33 ± 5.77a	26.64 ± 2.95a	5.35 ± 0.06bc

AN, AP, AK, OM, pH represent available nitrogen, available phosphorus, organic matter, available potassium, pH value.

*CK* the rice monoculture field, *RT3* the planting area in the 3rd year of rice-turtle field, *RT6* the planting area in the 6th year of rice-turtle field, *RT9* the planting area in the 9th year of rice-turtle field.

Different letters within columns indicate significant differences at P < 0.05.

### Soil bacterial community diversity

**Figure 1:**
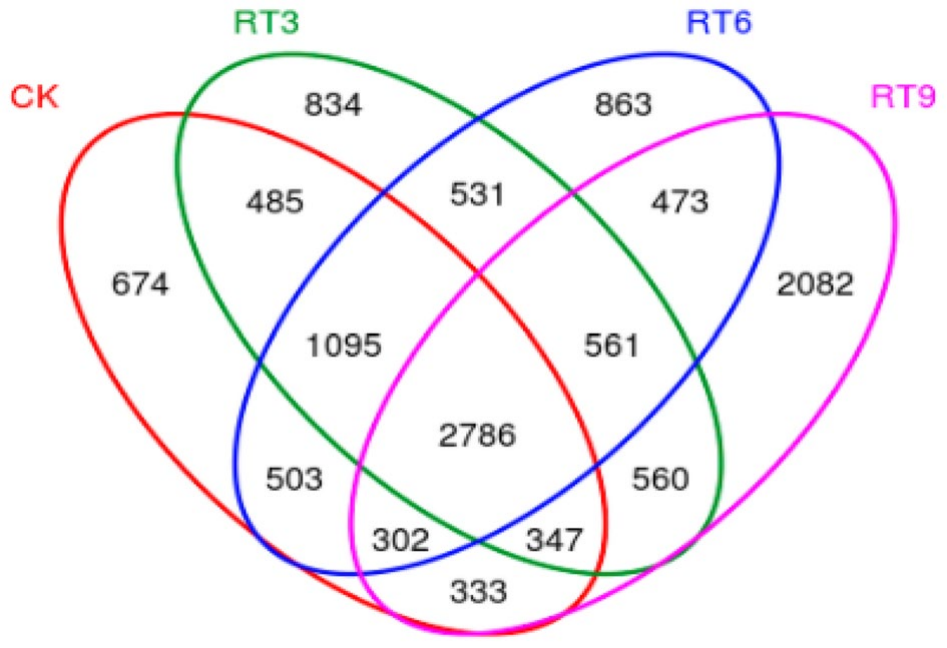
Venn diagram of soil bacterial community. *CK* the rice monoculture field, *RT3* the planting area in the 3rd year of rice-turtle field, *RT6* the planting area in the 6th year of rice-turtle field, *RT9* the planting area in the 9th year of rice-turtle field.

**Figure 2:**
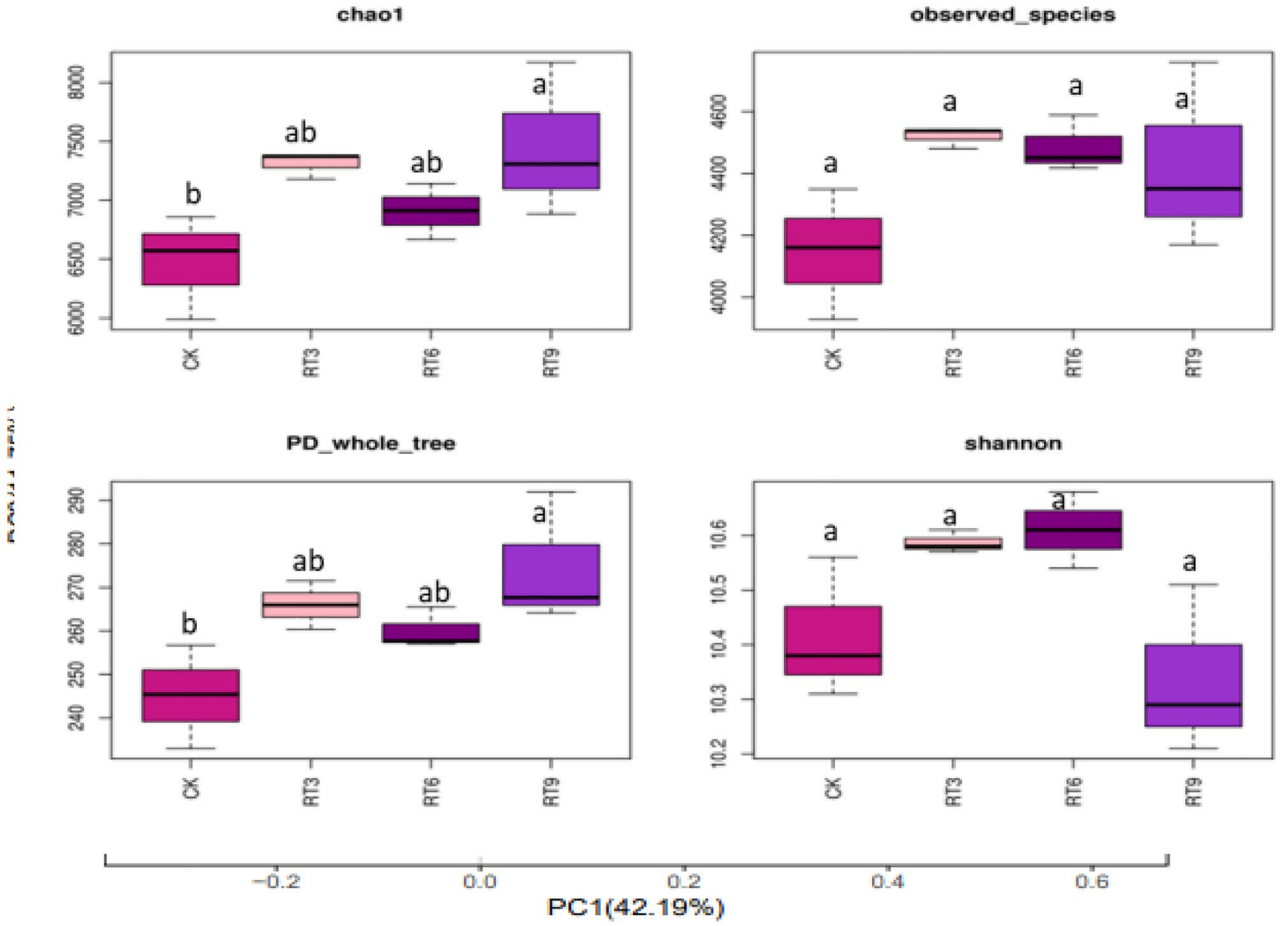
Bacterial diversity index of rice field soil in different riceturtle symbiosis fields. *CK* the rice monoculture field, *RT3* the planting area in the 3rd year of rice-turtle field, *RT6* the planting area in the 6th year of rice-turtle field, *RT9* the planting area in the 9th year of rice-turtle field. Different letters indicate significant differences at P < 0.05.

**Figure 3:**
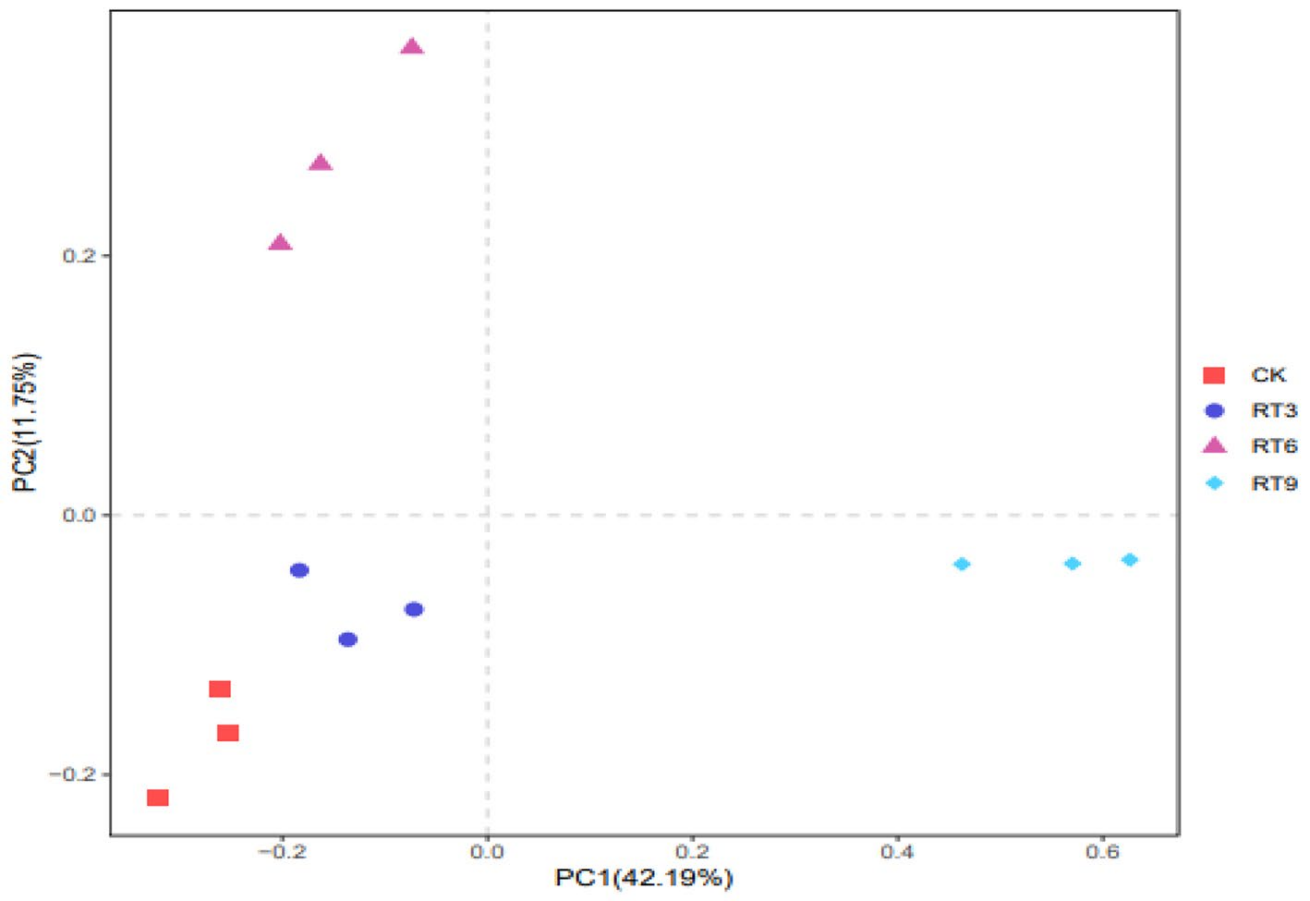
PCoA analysis of soil bacteria community under different rice-turtle coculture fields. *CK* the rice monoculture field, *RT3* the planting area in the 3rd year of rice-turtle field, *RT6* the planting area in the 6th year of rice-turtle field, *RT9* the planting area in the 9th year of rice-turtle field.

Clustering analysis of OTU on the sample sequences at the 97% similarity level was conducted to obtain a Venn diagram (Fig. 1). A total of 12,429 OTUs were identified in 4 treatments, there were 2786 OTUs common among them, and the maximum number of unique OTUs was observed in RT9. The Alpha diversity index of soil bacterial community (Fig. 2) showed that the Chao1 index and PD-whole-tree index of RT9 treatments were significantly higher than those of CK. but there was no significant difference in the observed species and the Shannon index among treatments. Principal coordinate analysis (PCoA) can compare the similarity of species composition between different treatments. The two principal coordinates explained 53.94% of the difference, and the sample points in each treatment group were relatively concentrated (Fig. 3). The sample points under CK and RT3 treatments were the closest and in the third quadrant, indicating that the soil bacterial community structure of these two treatments was similar. However, the bacterial community composition differed distinctly among rice-turtle coculture systems with different planting years.

### Soil bacterial community structure

**Figure 4:**
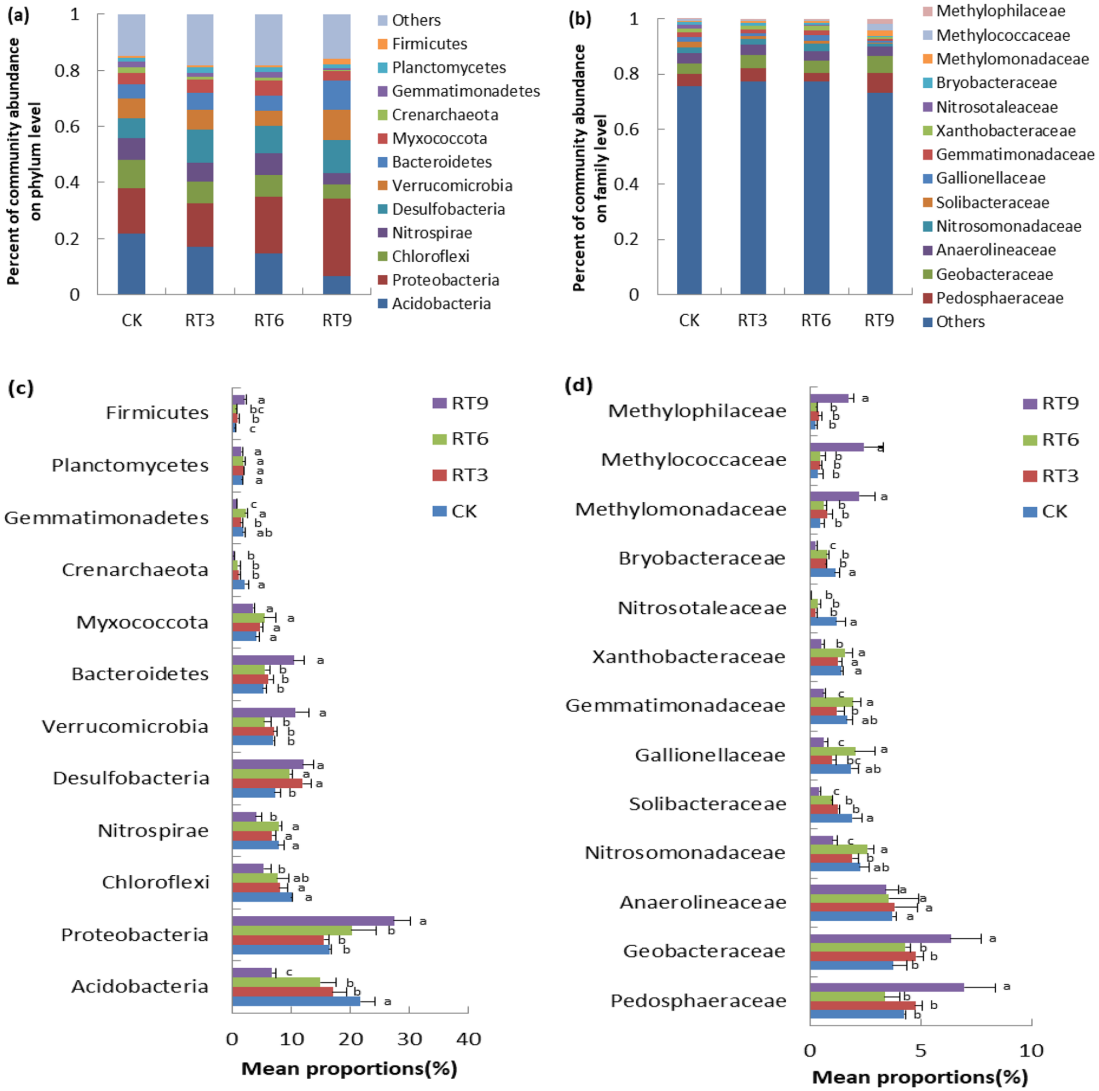
The average relative abundances on phylum (**a,c**) and family (**b,d**) level of soil bacterial communities under different rice-turtle co-culture fields. *CK* the rice monoculture field, *RT3* the planting area in the 3rd year of rice-turtle field, *RT6* the planting area in the 6th year of rice-turtle field, *RT9* the planting area in the 9th year of rice-turtle field. Different letters indicate significant differences at P < 0.05.

The sequencing results showed that 8 phyla and 196 families were newly generated in the topsoil bacterial community of the rice-turtle coculture system compared with the control. At the phylum level (Fig. 4a,c), the relative abundance of *Acidobacteria, Desulfobacteria* and *Crenarchaeota* were significantly different among treatments. The dominant bacterial phyla across different treatments were *Proteobacteria* (15.52–27.39%), followed by *Acidobacteria* (6.71–21.69%). With the increase of the planting years of rice-turtle coculture system, the relative abundance of *Acidobacteria, Chloroflexi, Nitrospirae* and Crenarchaeota decreased, while the relative abundance of *Proteobacteria, Desulfobacteria, Verrucomicrobia, Bacteroidetes* and *Firmicutes* increased. For example, *Acidobacteria* abundance greatly decreased from 21.69% in CK to 6.71% in RT9, while *Proteobacteria* abundance greatly increased from 16.38% in CK to 27.38% in RT9, and *Desulfobacteria* abundance greatly increased from 7.20% in CK to 12.00% in RT9. At the family level (Fig. 4b,d),* t*he dominant bacterial family across different treatments were *Pedosphaeraceae* (3.37–6.97%), *Geobacteraceae* (3.75–6.34%) and *Anaerolineaceae* (3.42–3.82%). Among them, the relative abundance of *Pedosphaeraceae* and *Geobacteraceae* was the highest in RT9 treatment. The relative abundance of *Methylomonadaceae*, *Methylococcaceae* and *Methylophilaceae* (1.74–2.44%) in RT9 was significantly higher than that of other treatments, and the relative abundance of *Nitrosotaleaceae* and *Gallionellaceae* treated with rice-turtle coculture system was significantly lower than that of CK.

**Figure 5:**
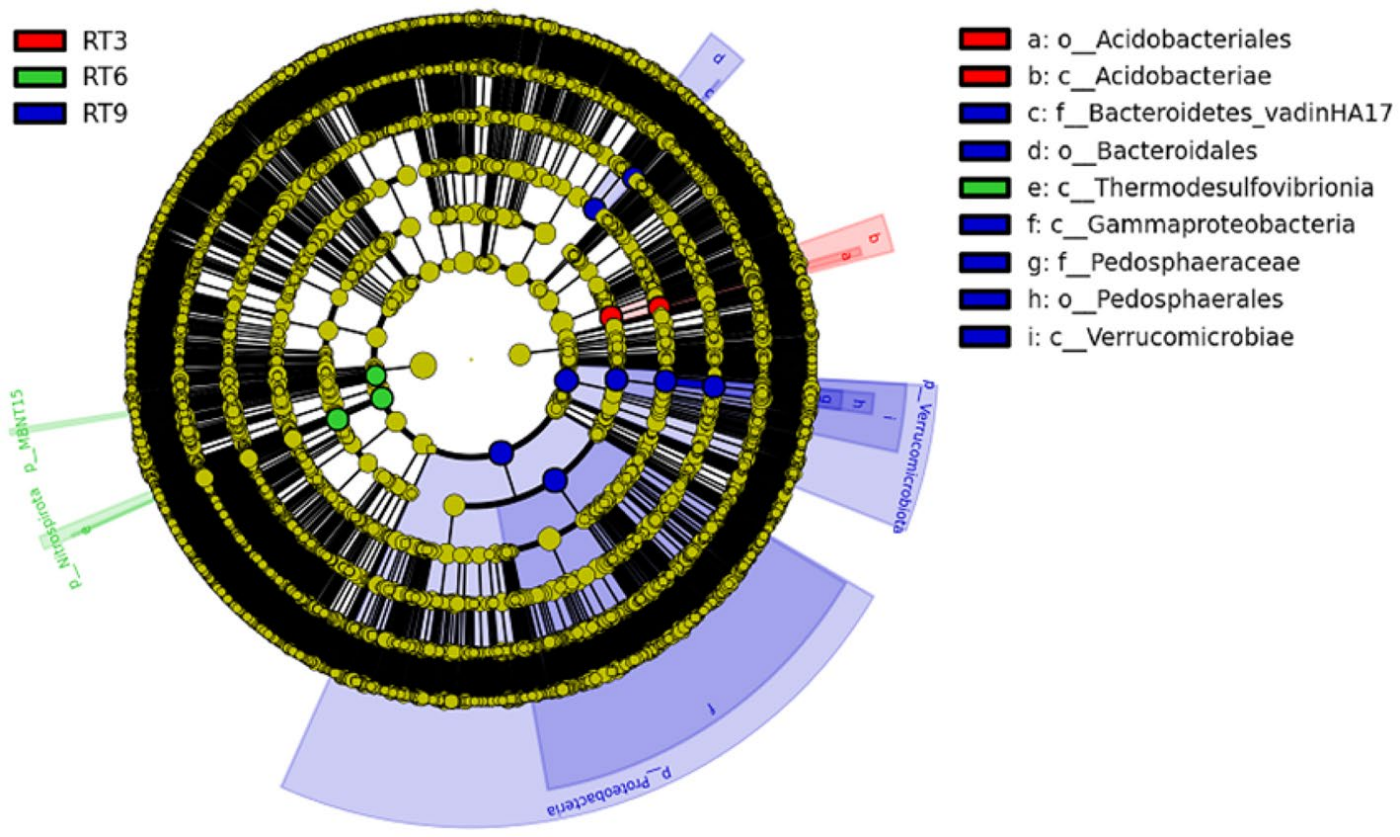
LEf Se analysis of soil bacteria community (LDA ≥ 4.0). *CK* the rice monoculture field, *RT3* the planting area in the 3rd year of rice-turtle field, *RT6* the planting area in the 6th year of rice-turtle field, *RT9* the planting area in the 9th year of rice-turtle field.

LEf Se analysis (Fig. 5) showed that with the increase of planting years in the rice-turtle coculture systems, the number of significant difference groups of bacteria showed an increasing trend. The significant difference groups of RT3, RT6 and RT9 treatments were 2, 3 and 8, respectively. At the phylum level, the significant difference groups of RT9 treatment were *Proteobacteria* and *Verrucomicrobia*, the significant difference groups of RT6 treatment were *Nitrospirae* and *MBNT15.*

### Comparative analysis of soil bacterial community

**Figure 6:**
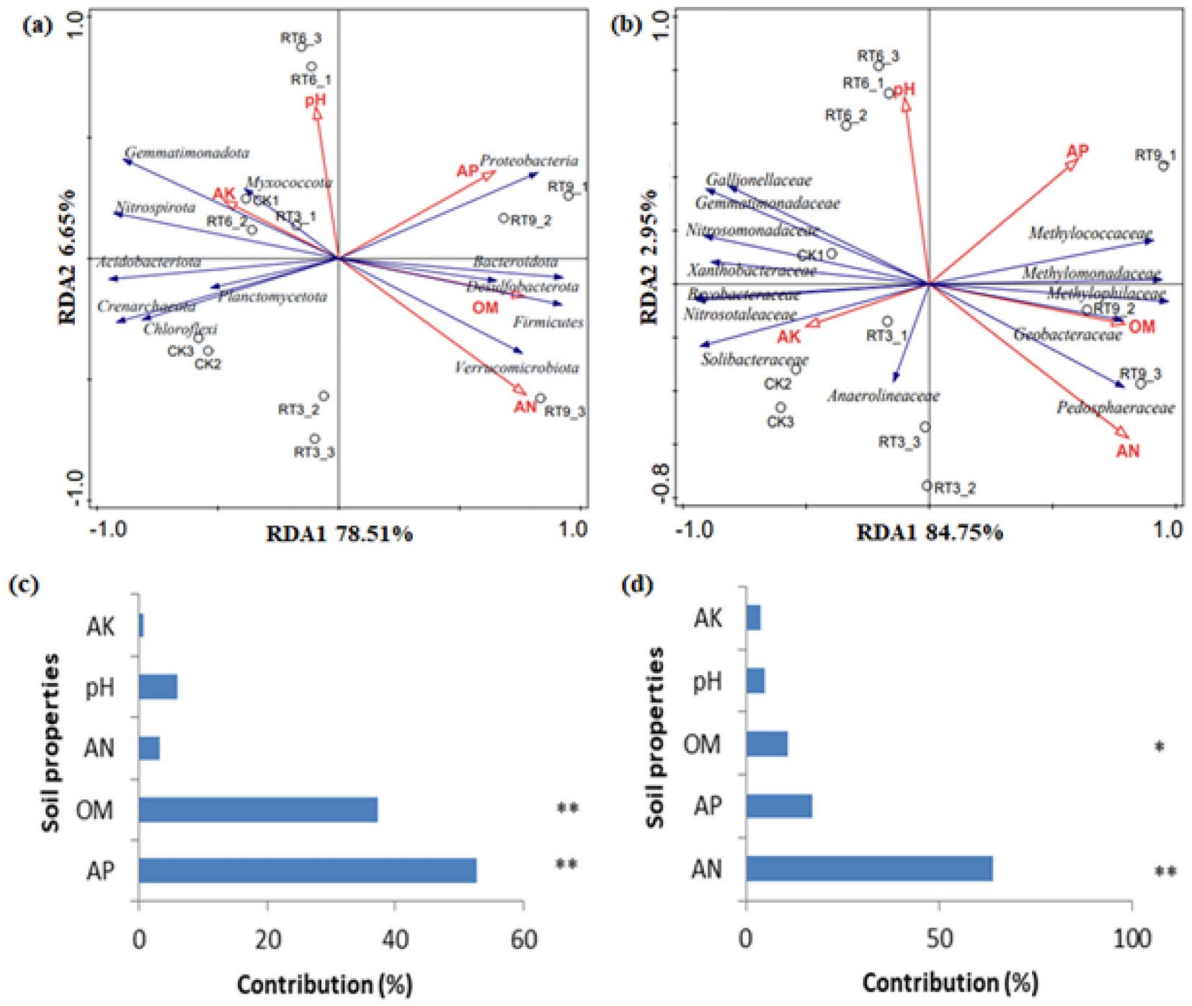
The RDA (Redundancy analysis) of soil fertility parameters and dominant phyla (**a,c**) and family (**b,d**) in paddy field soil for rice-turtle coculture system. *CK* the rice monoculture field, *RT3* the planting area in the 3rd year of rice-turtle field, *RT6* the planting area in the 6th year of rice-turtle field, *RT9* the planting area in the 9th year of rice-turtle field. *Indicates 0.01 < P ≤ 0.05, **indicates 0.001 < P ≤ 0.01.

The results of RDA analysis showed that the soil properties together explained 85.16% of the total variations in bacterial community composition at the phylum level, and 87.70% at the family level (Fig. 6a,b). AP was the main factor affecting the community structure of soil bacteria at the phylum level, while AN was the main factor affecting the community structure of soil bacteria at the family level. At the phylum level, *Proteobacteria* was positively correlated with AP, *Bacteroidota, Desulfobacterot* and *Firmicutes* were positively correlated with OM, and *Verrucomicrobiota* was positively correlated with AN. At the family level, *Methylomonadaceae, Methylococcaceae*, *Methylophilaceae* were significantly positively correlated with OM. The composition of soil bacterial community was significantly correlated with OM (Fig. 6c,d).

## Discussion

Soil organic matter and available nitrogen, phosphorus and potassium content are important indicators for evaluating soil quality. Previous studies have shown that rice–fish integrated farming could improve the content of soil organic matter and available nutrients, especially the content of soil total nitrogen and alkali-hydrolyzable nitrogen^14,15^. In this study, the AN, AP and OM contents of RT9 treatment were 25.40%, 51.11% and 23.33% higher than CK, respectively. Xiao et al.^16^ also showed that the contents of soil organic matter, alkali-hydrolyzable nitrogen, available phosphorus and available potassium were significantly increased by 17.82%, 13.80% and 37.37% (P < 0.05) in the rice-turtle coculture system. But Zhao et al.^13^ showed that there was no significantly difference in soil organic matter content between rice–fish integrated farming and rice monoculture, which may be related to the capture of fish before the rice harvest. Wu et al.^17^ found that the turtle stocking density affected the soil organic matter, soil organic matter content increased under high stocking density. The residual feed and excreta of cultured animals in the rice-turtle coculture system were decomposed into nutrients under the action of microorganisms. The number of adult turtles were increased after 9 planting years, and the food intake and excretion of turtles also increased. So RT9 increased the turtle stocking density, which ultimately caused the residual bait and excrement released more nutrients after microbial decomposition. Our study showed that the soil pH of the rice-turtle coculture systems was higher than that of CK, which was consistent with the results of Wu et al.^17^, which may be related to the lower fertilizer nitrogen input in the rice-turtle co-culture systems. This indicated that the rice-turtle coculture system was beneficial to improve the pH value of acidic soil.

The soil microbial diversity, a biomarker indicator of soil health^18^, which is affected by many factors such as tillage methods, planting patterns, water and fertilizer management, is especially sensitive to changes in soil physical and chemical properties and soil environment. For the rice-turtle coculture system, the feeding and excretion activities of turtles have a corresponding impact on the nutrient cycling and energy flow of the paddy ecosystem. The rice-turtle coculture systems created a flooded environment, and the activity of turtles changed the microenvironment of surface soil in the field, which ultimately caused the changes in soil microbial community. The results showed that 8 phyla and 196 families were newly generated in the topsoil bacterial community of the rice-turtle coculture system compared with the control, and the rice-turtle coculture system could improve the diversity of bacterial communities in topsoil. This could be attributed to the amphibious characteristics of turtles, and the activity range of turtles was the whole rice area. Li et al.^10^ also found that 12-year rice-crayfish-turtle farming led to much higher soil microbial community diversity. But Wu et al.^17^ found that the soil microbial diversity was highest in rice monoculture farming due to the greater fertilizer input in the rice monoculture group than in all co-culture groups. PCoA analysis showed that the soil bacterial community structure of RT6 and RT9 was significantly different from that of RT3, which was similar to Ding et al.^19^. However, the soil bacterial community structure of RT3 was similar to that of CK. This finding means that with the increase of planting years, the differentiation of the soil bacterial communities in the rice-turtle fields was more significant, which may be related to the activity intensity of turtles in the field.

Microbial community composition is sensitive to the change of soil environment, and it has important effects on organic matter dynamics and nutrient cycling^20^. The field transformation and management measures of the rice-turtle coculture system changed the field environment. The activities of the turtle in the field has large effects on the topsoil environment, which changed the microbial community structure of the topsoil. In this study, *Proteobacteria* and *Acidobacteria* the common dominant phylum in the four treatments, *Proteobacteria* is a kind of eutrophic bacteria, which live in a nutrient-rich environment and mainly decompose unstable carbon pools in the soil surface, closely related to the decomposition of organic matter^21^. *Acidobacteria* is a kind of oligotrophic bacteria, which is negatively correlated with soil nitrogen application, and mainly decompose the deep soil carbon pool which is difficult to degrade^22^. The results showed that *Acidobacteria* abundance greatly decreased from 21.69% in CK to 6.71% in RT9, while *Proteobacteria* abundance greatly increased from 16.38% in CK to 27.38% in RT9. Because the higher AN, AP and OM contents in RT9 provides sufficient metabolic substrates, promotes the growth and reproduction of microorganisms. LEf Se analysis also showed that *Proteobacteria* was a significant difference group in RT9 treatment. The number of significant difference groups increased with the increase of planting years consistent with the results of pot experiment^19^. Another interesting result of this study is that the relative abundance of *Methylomonadaceae, Methylococcaceae* and *Methylophilaceae* (1.74–2.44%) in RT9 treatment was significantly higher than that in other treatments. These three families belong to *Proteobacteria* and are mostly methane-oxidizing bacteria, which can promote methane oxidation^23^. Chen et al.^24^ showed that the abundances of methanogen and methanotroph in rice-fish coculture were significantly higher than those in rice monoculture. Compared with the rice monoculture system, the rice-fish coculture system may provide more nutrients and suitable growth environment for methanogens and methanotrophs, thereby increasing their abundance in the soil. It may be related to paddy field flooding and high soil organic matter. However, Bhattacharyya et al.^25^ showed that the emission of CH_4_ was significantly higher by 26% under rice-fish co-culture compared to rice monoculture. The results showed that the highest soil AN, AP, OM content was observed in RT9, it indicated that rice-turtle coculture systems in 9th year has the risk of increasing methane emissions.

The residual bait and excreta in rice-turtle system increased the content of soil organic matter. Previous studies have indicated that organic matter was significantly correlated with soil bacterial community structure^26^. In this study, RDA analysis showed that AP and AN were the main factors affecting the community structure of soil bacteria, and the composition of soil bacterial community was significantly correlated with OM. In addition, *Methylomonadaceae, Methylococcaceae* and *Methylophilaceae* were positively correlated with OM. Xia et al.^27^ found that the relative abundance of methane-oxidizing bacteria was significantly positively correlated with dissolved organic carbon content.

There were still some flaws in the study, for example, Microbial properties were determined in fresh soil, while physicochemical properties were determined in air-dried soil. The CK soil sample was collected from an aerobic environment, while the treated soil (rice-turtle coculture systems) was collected from an anaerobic environment, which may mask the influence of soil moisture, pH and other key factors on soil microorganisms.

## Conclusions

The content of AN and AP were the main factors affecting the composition of soil bacterial community. The rice-turtle coculture systems with 9 planting years could significantly increase AN, AP and OM contents, improve the relative abundance of *Methylomonadaceae, Methylococcaceae* and *Methylophilaceae*. However, this study is only a preliminary exploration of the basic characteristics of soil bacterial community composition and its relationship with soil properties in rice-turtle coculture system. The relationship between key functional microbial methanotrophs and methane emissions from paddy fields needs to be further studied.

## Materials and methods

### Experimental field description and experimental design

**Figure 7:**
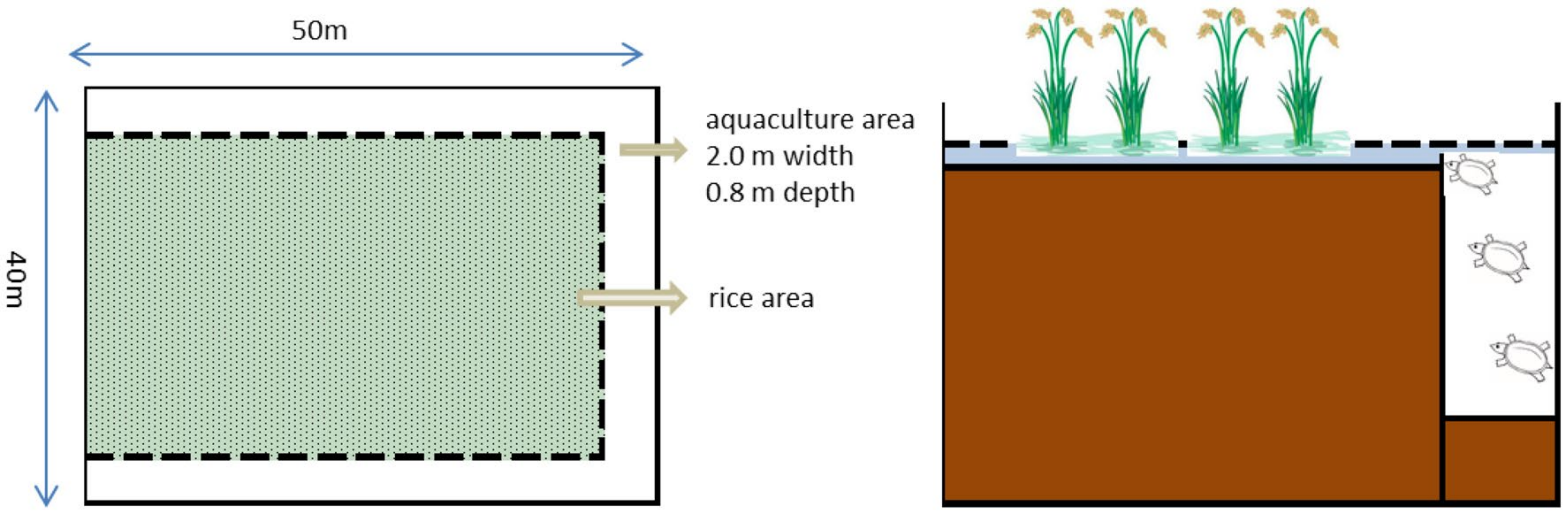
Layout diagram of the rice turtle co-culture system.

The experimental site is located in Kongpuzhong Family Farm, Jinshi Village, Dahu Town, Liuyang City, Hunan Province, China (28° 22′ 37″ N, 113° 53′ 46″ E). This area has a subtropical humid monsoon climate, with an annual average temperature of 16–18 °C and annual precipitation of 1350 mm. The soil type in this region is gley paddy soil, and the rice variety is Nongxiang 32. In 2013, the farm started operations with Chinese soft-shelled turtle as the main fish species cultured in paddy fields. Moreover, 150 grass carps per hectare were farmed to control weeds in the field, and small fish, shrimp, field snails were farmed in the ditches as food for turtles. Four paddy fields were selected to conduct experimental studies, including (1) *CK* the rice monoculture field; (2) *RT3* the planting area in the 3rd year of rice-turtle field; (3) *RT6* the planting area in the 6th year of rice-turtle field; (4) *RT9* the planting area in the 9th year of rice-turtle field. Rice is planted from May to October every year, shallow flooding irrigation was adopted during the rice season, the rice field is drained for 7 days in rice tillering stage and yellow ripe stage, respectively. The field surface is flooded by more than 30 cm to inhibit the growth of weeds and provide the turtles more living space in rice-turtle fields during the rest time, and oil rape is planted after rice harvest in monoculture system. Rice-turtle fields were applied with 375 kg ha^−1^ of compound fertilizer (15% N, 15% P and 15% K) as the basal fertilizer, and no agrochemical inputs throughout the year except for base fertilizer. Rice monoculture field was applied with 375 kg ha^−1^ of compound fertilizer (15% N, 15% P and 15% K) as the basal fertilizer, and 75 kg ha^−1^ of urea (46% N) was used as topdressing at the tillering stages. The initial density of Juvenile soft shelled turtle was 7500/hm^2^, and the activity range of turtles was the whole rice area. Turtles feed on small fish, shrimp, snails and other small animals in the field, the artificial feed was mainly Pomacea canaliculata and animal viscera. The layout (Fig. 7) of the rice-turtle field (40.0 m × 50.0 m) consisted of a rice planting area (37.5 m × 45.0 m) and surrounded on three sides by an aquaculture ditch (0.8 m in depth and 2.0 m in width). The aquaculture area accounts for about 10% of the paddy field area.

### Soil sampling and measurements

After rice harvest in October 2021, soil samples were collected from the top-layer (0–20 cm) of the 4 designed treatments. The soil samples collected from 5 points were mixed into one sample by S-type sampling method. Three duplicates were collected for each treatment and 12 soil samples were collected in total. All samples were separated into two parts after removing impurities and mixing well: one part was brought back to laboratory immediately in a cold storage box for the extraction of soil microbial DNA, and the other part was air dried to determine soil physicochemical properties. AN was determined by the alkaline hydrolysis diffusion method, AP was extracted using 0.5 mol⋅L^−1^ NaHCO_3_, followed by the colorimetric measurement of inorganic phosphorus, using the molybdate-ascorbic acid method, and AK was determined by sodium tetraphe­nylboron turbidimetry, following extraction with 1 mol⋅L^−1^ neutral ammonium acetate. Soil pH was determined in 2:5 (w/v) soil: water suspen­sions with a calibrated pH meter (Mettler-Toledo FE 20, Zurich, Switzerland). OM was determined by the potassium dichromate oxidation–external heating method.

DNA extraction, PCR amplification and MiSeq Sequencing were entrusted to Beijing Aowesen Gene Technology Co. The CTAB method was used to extract the total DNA of the sample, and the extracted genomic DNA was detected by 1% agarose gel electrophoresis. The DNA was purified with Agencourt AMPure XP Nucleic Acid Purification Kit and quantified by an ultraviolet spectrophotometer. The PCR amplification used Q5 high-fidelity DNA polymerase (NEB, Ipswich, MA, USA), and the V3–V4 region of the 16S rDNA genes were amplified using the primers 338F (ACTCCTACGGGAGGCAGCA) and 806R (GGACTACHVGGGTWTCTAAT)^28^. A total of 20 pM DNA for each sample were pooled and sequenced in an Illumina MiSeq platform (Illumina, SanDiego, CA, USA) with a 600-cycle kit (2 × 300 bp paired ends).

### Data processing and analysis

The raw sequence dataset was analyzed with QIIME (Version 1.8.0). Sequences were removed if the read length was < 120 bp, with a mean quality score < 20. Sequences with ≥ 97% similarity were assigned to the same operational taxonomic units (OTUs) using UPARSE. The RDP Classifier (v16) was used to annotate taxonomic information. Redundancy analysis (RDA) was carried out to explore the relationship between soil properties and bacterial community composition using Conoco 5. The microbial communities were clustered by using principal coordinates analysis (PCoA) based on the unweighted UniFrac distance and Bray–Curtis distance matrix. In addition, ANOSIM test, Kruskal–Wallis test, Wilcoxon tests and Metastats test were employed to quantify the statistical differences among treatments. Data were compared using analysis of variance (ANOVA) in IBM SPSS 23.0 software (SPSS Inc., USA). Alpha diversity (Chao1 and Shannon index) was calculated with QIIME (Version 1.8.0). Pearson’s correlation coefficients between soil properties.

## Data Availability

The data that support the findings of this study are available from the corresponding author upon reasonable request.
